# Impact of Social Isolation during the COVID-19 Pandemic on Mental Health, Substance Use, and Homelessness: Qualitative Interviews with Behavioral Health Providers

**DOI:** 10.3390/ijerph191912120

**Published:** 2022-09-25

**Authors:** Alexiss Jeffers, Ashley A. Meehan, Jordan Barker, Alice Asher, Martha P. Montgomery, Greg Bautista, Colleen M. Ray, Rebecca L. Laws, Victoria L. Fields, Lakshmi Radhakrishnan, Susan Cha, Aleta Christensen, Brandi Dupervil, Jorge V. Verlenden, Cynthia H. Cassell, Alaina Boyer, Barbara DiPietro, Margaret Cary, Maria Yang, Emily Mosites, Ruthanne Marcus

**Affiliations:** 1 Centers for Disease Control and Prevention COVID-19 Emergency Response, 1600 Clifton Rd., Atlanta, GA 30329, USA; 2 Oak Ridge Institute for Science and Education (ORISE) Fellow, Oak Ridge Associated Universities, 100 Orau Way, Oak Ridge, TN 37830, USA; 3 National Healthcare for the Homeless Council, 604 Gallatin Ave, Nashville, TN 37206, USA; 4 Oregon Health Authority, 500 Summer Street NE, Salem, OR 97301, USA; 5 Downtown Emergency Service Center, 515 3rd Ave, Seattle, WA 98114, USA

**Keywords:** COVID-19, social isolation, behavioral health providers, mental health, substance use, homelessness

## Abstract

The United States is experiencing a syndemic of homelessness, substance use disorder, and mental health conditions, which has been further exacerbated by the COVID-19 pandemic. Although it is expected that mitigation strategies will curb community transmission of COVID-19, the unintended consequences of social isolation on mental health and substance use are a growing public health concern. Awareness of changing mental health and substance use treatment needs due to the pandemic is critical to understanding what additional services and support are needed during and post-pandemic, particularly among people experiencing homelessness who have pre-existing serious mental illness or substance use disorder. To evaluate these effects and support our understanding of mental health and substance use outcomes of the COVID-19 pandemic, we conducted a qualitative study where behavioral health providers serving people experiencing homelessness described the impact of COVID-19 among their clients throughout the United States. Behavioral health providers shared that experiencing social isolation worsened mental health conditions and caused some people to return to substance use and fatally overdose. However, some changes initiated during the pandemic resulted in positive outcomes, such as increased client willingness to discuss mental health topics. Our findings provide additional evidence that the social isolation experienced during the pandemic has been detrimental to mental health and substance use outcomes, especially for people experiencing homelessness.

## 1. Introduction

The World Health Organization (WHO) declared coronavirus 2019 (COVID-19) a pandemic on 11 March 2020, after severe acute respiratory syndrome coronavirus 2 (SARS-CoV-2) rapidly spread across the world [1,2]. To curb viral spread, community mitigation strategies like sheltering-in-place, mask mandates, and physical distancing became critical. These strategies resulted in unprecedented changes worldwide.

In addition to the COVID-19 pandemic, the United States has been experiencing a syndemic of homelessness, substance use disorder (SUD), and mental health conditions. In 2019, approximately 567,715 people were experiencing homelessness, 70,000 overdose deaths occurred, and 51.5 million people were experiencing mental illness [3,4,5]. Among people experiencing homelessness (PEH), roughly 20% reported severe mental illness and 15% reported chronic substance use [3].

Although vaccination and mitigation strategies will eventually curb community transmission of COVID-19, the unintended consequences of asking people to remain in isolation on mental health and substance use are a growing public health concern [6]. Since the onset of the COVID-19 pandemic, increases in anxiety, depression, and post-traumatic stress disorder have been reported globally [7,8]. A systematic, rapid review of the psychological impact of quarantine found that pandemic-related stressors might have long-lasting effects on well-being [9]. Social isolation, as a result of recommended COVID-19 mitigation strategies, has been associated with exacerbation of mental health challenges and overdose deaths [10,11,12]. People with SUD have disproportionately elevated rates of mental health challenges; those with a previous mental health diagnosis reported greater exacerbations of psychological symptoms during the pandemic than those who did not report previous diagnoses [12,13].

Awareness of changing mental health and substance use treatment needs due to the pandemic is critical to understanding what additional services and support are needed during and post-pandemic, particularly among PEH with pre-existing serious mental illness (SMI) and pre-existing SUD. The aim of this study was to describe behavioral health providers perceptions of mental health and substance use outcomes for PEH during the COVID-19 pandemic. To achieve this aim, we conducted a descriptive qualitative study with behavioral health providers across the United States.

## 2. Materials and Methods

### 2.1. Participants and Recruitment

Standardized, in-depth interviews were conducted from August 20 through 14 October 2020 with 50 behavioral health providers across the United States who provide services to clients experiencing homelessness who have SMI or SUD. Purposive sampling was used to identify study participants who routinely work with PEH and were from different geographic regions of the country. A study team from the Centers for Disease Control and Prevention (CDC) collaborated with the National Health Care for the Homeless Council (NHCHC) to identify and recruit potential participants. NHCHC distributed an invitation to participate in a qualitative research study to member organizations on an email listserv. Interested participants contacted the CDC study team, who then determined eligibility for participation. Those who worked in facilities serving PEH but were not directly involved in providing services to clients were excluded from participation; we sought to understand the perspectives of client-facing providers. Participants were provided a $100 gift card in appreciation for their time and participation.

### 2.2. Data Collection Tools and Procedures

CDC team members served as interviewers and notetakers. Interviewers and notetakers completed an internal training on qualitative data collection approaches and techniques prior to data collection. The two-hour training was conducted by a doctoral-level medical anthropologist and discussed the goals and paradigm in qualitative data collection, as well as technical best practices for conducting interviews and note taking.

Participants were invited to complete a brief Research Electronic Capture (REDCap) survey prior to or during the phone interview. The survey included questions about provider demographics and role and facility characteristics (staffing, client characteristics, and behavioral health services provided). Interviews were conducted by phone using a standardized, semi-structured interview developed by CDC, with input from NHCHC and behavioral health providers (Supplemental File S1). Each interview began with a review of participant rights; all were allowed to skip questions or withdraw at any point, the purpose of data collection and planned use and protection of data and personal data, and risks and benefits were explained. At the end of the consent script, interviewers asked for verbal indication of consent to participate, before asking permission to audio record the conversations.

On average, interviews took 45 min to complete, and covered topics such as changes to service provision during COVID-19, perspectives of changes to mental health and substance use among their clients, and about success and unintended benefits that emerged during COVID-19.

### 2.3. Data Analysis

Interviews were audio recorded and transcribed verbatim by CaptionSync, a transcription company. Transcripts were imported into MAXQDA, a qualitative data management software, for analysis [14]. Because the authors sought to describe the perspectives and experiences of participants without generating new theory, thematic analysis was conducted using Braun & Clarke’s six phase framework for analysis: (1) become familiar with the data (2) generate initial codes (3) search for themes (4) review themes (5) define themes (6) write-up themes [15]. The coding team reviewed interview transcripts and interview notes to become familiar with the data, and an a priori codebook was developed based on the interview guides and preliminary review of the data. Three members of the project team (AJ, AM, JB) each coded one transcript independently to assess consistent application of codes. The rest of the transcripts were coded independently by two of the three coders, with regular check-ins to discuss new codes that should be added or challenges determining code application. Codes were then grouped by topic to create themes, described in the results.

## 3. Results

### Study Population

Table 1 describes characteristics of the 50 behavioral health providers from across all 10 Department of Health and Human Services’ regions in the United States. No participants withdrew their participation during or after their interview. Most participants were female (*n* = 42, 84%) and non-Hispanic White (*n* = 31, 62%). Behavioral health providers interviewed included case managers (*n* = 10), social workers (*n* = 8), therapists or counselors (*n* = 7), nurse or nurse practitioners (*n* = 6), outreach staff (*n* = 5), other behavioral health providers (*n* = 5), directors (*n* = 5), peer specialists (*n* = 3), and a psychiatrist or psychologist (*n* = 1) who provide behavioral health services including psychiatric or mental health evaluations, treatment, or referral; substance use evaluations, treatment, referral, provision of medications for opioid use disorder (MOUD); counseling; non-pharmacologic treatments; or substance use disorder-related services. All participants provided services to clients with a behavioral health-related diagnosis, 98% served PEH and clients with SUD, and 88% served clients with SMI. Since these conditions are intersectional, 96% of providers served PEH clients with SUD, 98% served PEH clients with a behavioral health diagnosis, and 88% served PEH clients with a SMI. Most of the participants worked in community health centers (*n* = 32, 64%), served on street outreach teams (*n* = 17, 34%), or delivered out-patient psychiatric services (*n* = 11, 22%).

## 4. Themes

### 4.1. The Negative Impacts of Social Isolation

The detrimental impact of social isolation emerged as a cross-cutting theme (Table 2). Providers expressed that prior to the COVID-19 pandemic, their clients depended on social connection, in-person services and groups, and access to resources to maintain recovery for their SUD or mental health condition. Social isolation was, “the worst thing for anybody with mental health or substance abuse” conditions. One provider noted that social isolation was a risk factor for their clients, “because the opposite of addiction is connection.” Providers reported some clients chose to risk COVID-19 exposure rather than stay isolated.

*That’s probably the most prominent, the isolation. Some people would rather risk exposure than be isolated. That’s been pretty common across the board about most of the people I’ve talked to. One of the biggest issues they’re experiencing is the isolation, lack of community, and connection.*

Social isolation had a largely negative, pervasive impact on the overall wellbeing of their clients and was a catalyst for negative outcomes, including return to substance use, increased substance use, fatal and non-fatal overdose, and an exacerbation of mental health symptoms.

### 4.2. Return to Substance Use and Increased Substance Use

Providers reported that people with SUDs who maintain abstinence in recovery thrive on a consistent routine of multi-faceted treatment, including group counseling, individual therapy, and MOUD (where appropriate). During the COVID-19 pandemic, social isolation led to a disruption of routine and structure, boredom, and lack of connection for many. Providers described that because social structures are embedded into many recovery-oriented programs, when this form of connection was no longer available, many clients experienced a dangerous catalyst toward returning to substance use. One provider pointed out,

*They don’t have that interaction that they had before. Before we had COVID-19, we had medication assisted treatment, as well as groups, and more access physically to their counselor. So that one-two combination, it’s proven successful in helping people in their recovery. They don’t have that now.*

Providers reported feeling the impact of recovery support groups such as Alcoholics Anonymous (AA) or Narcotics Anonymous (NA), was lessened as they moved online, and reported that many of their clients did not like the virtual format. Providers also reported the importance of facilitating groups in-person because they could better assess how their clients were doing, and felt their clients enjoyed connecting with others who were also in treatment and were positively influenced by others also in recovery.

Providers reported that social isolation fueled chaotic, increased use of substances among clients. The pandemic magnified many of the issues that providers reported their clients experienced, including mental illness, SUD, and homelessness. One provider described,

*I think that relapse is a big thing. You spend 10 years of doing a specific drug, or way of life or living or several drugs, and then trying to get off and then based off of the world shifting in such, what I call madness, it kind of gives you a reason to relapse and go backwards. So that’s been a big issue with relapsing. It’s an up and down and up and down thing, stronger than it already has been because our clients are facing homelessness, addiction, and mental health. So, relapse is a big thing, and out here on the streets, drugs are, clearly they’re here. But I feel like a wave of more [drugs] have come in, so it’s been kind of chaotic.*

Providers shared that their clients reported the pandemic and subsequent social isolation made them feel “out of control”, which led to increased substance use. Additionally, the instability of the COVID-19 pandemic caused clients to report purchasing more drugs than usual, out of concern they may not have access again. Because of this, “people are tending to use in a little bit more risky way than they had been,” increasing the likelihood of overdose.

### 4.3. Changes in Overdose Risk and Rates

Providers reported that clients with SUD who returned to substance use had to decide whether to use substances alone or risk contracting COVID-19 by using with others. One provider noted that “being alone, more people have overdosed” and “the messaging about need[ing] to isolate didn’t do a good job of taking the risks involved into account.” Isolation goes against the harm reduction strategy to ‘never use alone’ to reduce the risk of a fatal overdose. Another provider shared that using alone “is the exact opposite of what we need for an opioid epidemic.” As a result, providers described how social isolation could lead to fatal consequences. A provider shared,

*I think people are using more substances. I think they’re using alone. We have an increase of I think 50% more deaths in the homeless population due to overdosing. So there’s been some things that have been available to them that they never had before, and I think they’re also suffering in ways that they weren’t before.*

Another provider noted that more of their clients had died of overdose, whereas they did not know of any who died from COVID-19.

### 4.4. Access to Medications for Opioid Use Disorder (MOUD)

Providers and harm reductionists have advocated for greater access to MOUD, which treats opioid use disorder by preventing withdrawal symptoms and reducing cravings. During the COVID-19 pandemic, clinics and group sessions were closed, and some providers noted concern about maintaining client’s access to MOUD. Recognizing the need for ensuring access to medications, the Substance Abuse and Mental Health Services Administration (SAMHSA) posted COVID-19 guidance providing flexibility for allowing programs to offer MOUD for extended periods, ensuring that patients could access their prescriptions. Programs were also able to initiate buprenorphine treatment using telehealth and were able to see new patients without the usual requirement for an initial face-to-face visit, potentially lowering barriers to access. Providers considered this one of the positive impacts that the COVID-19 pandemic has had on their clients. However, they also noted it was not a perfect solution as providers were not evaluating their clients in-person as often to assess for signs of return to substance use.

*I think it’s the way we would provide medically assisted treatment in a shared medical visit setting. So, it would be in a group setting and we would kind of do a group accountability with a multi-disciplinary team. We’re not doing that anymore, so a lot of our patients are relapsing because they don’t have the social support component of the group modality. So that’s been really difficult for our patients. Although it’s increase[d] accessibility to Buprenorphine, it’s decreased the support services around it, so a lot more patients are relapsing.*

Providers reported that people receiving treatment for opioid use disorder benefit from a multi-modal approach, including support in group or individual therapy, or having a behavioral health provider to manage their care and treatment in addition to taking MOUD. Overall, however, providers described that they would like to see the reduced barriers to MOUD continue in the future.

### 4.5. Exacerbation of Mental Illness

Providers reported that the onset of the COVID-19 pandemic triggered anxiety, fear, depression, and stress in their clients. Subsequent social isolation due to stay-at-home orders detrimentally impacted client’s mental status because of the choice between staying home, which could exacerbate depression, or risking exposure by connecting with community. One provider shared, “if you have a serious mental illness, [isolation] seems to magnify whatever illness that person struggles with—depression, anxiety, psychosis—isolation is really detrimental.” For clients who had SMI, providers indicated paranoia as a common experience during the COVID-19 pandemic. One provider shared that among their clients,

*There’s a lot of skepticism, paranoia, [and] delusions. And so, there’s a lot of delusions all the time about being watched, or being targeted, or like the government is watching, or they’re listening, kind of things like that. And so, something like this really, really kicked that into high gear for a lot of clients.*

For clients experiencing unsheltered homelessness who were diagnosed with an SMI, feelings of paranoia intensified with the onset of COVID-19. PEH with SMI shared with providers that they felt that the pandemic was targeted against them “because they don’t want us around anymore.” Feelings of paranoia were especially pronounced when clients were being placed in emergency shelters and hotels, which were set up as protective housing or quarantine isolation sites. One provider described clients’ feelings of paranoia in the beginning of the pandemic while facilitating emergency sheltering:

*There has been such a feeling of fear and apprehension and mistrust that we’ve had to overcome to really let people know that we’re just here to try to take care of them. When the pandemic first—I’m thinking back to April and May [2020], we had the National Guard here with us at our emergency shelter and the hotel. And they were very helpful. But there was a great deal of fear seeing these soldiers at our sites. And I had people who were homeless tell me that they thought that we, meaning the service providers, were going to lock them all up in a concentration camp.*

However, providers described that the pandemic has sparked some positive change related to mental health outcomes. Providers described that more people than ever were reaching out for mental health support and that the COVID-19 pandemic had been the reason for people to seek help who may not have been willing to do so previously. Behavioral health providers noted that even though mental health was a stigmatized topic, more people were willing to talk about it because of COVID-19 and the many ways this experience has impacted their daily lives. Discussing mental health in general became more accepted and understood, and the conversations surrounding the impact of COVID-19 and isolation on mental health and substance use have become less stigmatized.

### 4.6. Navigating Homelessness While Experiencing Mental Illness or Substance Use Disorder

Providers had to navigate new COVID-19 restrictions for their clients experiencing homelessness, including restrictions that increased the difficulty of accessing limited shelter beds. Mitigation measures, including testing requirements, were very stressful for PEH. The additional stress of not being able to have a place to sleep without proof of a negative test, exacerbated feelings of depression.

*Some shelters started requiring that people got a COVID test or they couldn’t come back to the shelter. And that was really stressful for some because they didn’t know where to get the test. And they were so stressed out because they had nowhere to go. We had a guy crying in our lobby. He said, “I’m too old for this. I can’t stay on the street. I’m too old. And none of the shelters will take me back because I haven’t had the COVID test.” And he was just really, really depressed and distraught.*

Providers reported that the combined experience of homelessness and the emergence of a new, highly contagious virus was overwhelming, distressing, and confusing. For clients with a recent history of experiencing homelessness who were able to obtain housing, the transition into housing was made even more difficult by social isolation. One provider described “the socialness of homelessness” and how the new quietness of living alone exacerbated social isolation. Providers reported that their “housed clients are doing a better job of social distancing, but then experiencing higher levels of isolation, boredom, and depression”, which made it difficult for clients to cope with shelter-in-place orders. One provider noted feeling more worried about newly housed clients than sheltered clients,

*Because it’s different. Even if the sheltered life is not the best life, you have distractions, you have other people around that are friends, you have social things that you see, you have interactions with people. And when you’re alone, you’re just kind of alone with your thoughts and to think about things and reflecting on your life and look around and say like, “I have this thing I always wanted, but I just feel so alone because I’ve lived in this environment where there was always some kind of stimulation going on, and now I don’t have anything.” What do I do with that?*

Providers noted positive outcomes of the pandemic on homelessness, including the use of hotels as emergency shelters and “a small percent [of street homeless] were able to get in some temporary housing that would not have happened without COVID-19”. For another provider, they noted that one client’s mental health was positively impacted “because she was able to get into housing so quickly during this time”. Although some clients experienced reduced barriers to housing, there were also several that experienced increased barriers due to COVID-19 because landlords did not want to show apartments or their housing voucher expired. Although the use of hotels for emergency sheltering was temporary, it showed that solutions to combating the homelessness epidemic were possible.

## 5. Discussion

Understanding the mental health and substance use effects of the COVID-19 pandemic is critical to identify where future resources and interventions are needed to provide support. Behavioral health providers have unique knowledge of and expertise in the lived experiences of their clients, and the impact of homelessness, along with behavioral conditions like SUD and mental illness during the COVID-19 pandemic. These providers are most apt to discern changes among their clients and understand the impact of social isolation. Enhancing understanding of the unintended consequences of COVID-19 mitigation strategies seen by providers among their clients may help inform future public health interventions, policies and track long-term effects of the COVID-19 pandemic.

### Themes and Major Takeaways

Several important themes emerged from these interviews, including the far-reaching impact of COVID-19 mitigation measures and social isolation, which led to return to and increased substance use and overdoses. Prior to the COVID-19 pandemic, the United States was experiencing an opioid epidemic [16]. During the COVID-19 pandemic, research has demonstrated that COVID-19 related social isolation and the stress of living in a pandemic are correlated with return to use, increased substance use, and reduced access to SUD treatment services [17,18]. In fact, overdoses reached a record high and acceleration during the pandemic, with a 30.5% increase in the United States in 2020 according to provisional estimates as of 25 August 2021 [19].

The COVID-19 pandemic has also exacerbated feelings of depression, anxiety, and loneliness amidst social isolation. This finding is supported by previous research that COVID-19 mitigation measures, and subsequent effects of those measures including social isolation, may lead to mental health symptoms, loneliness, and psychological trauma [17]. Furthermore, the compounded stress of homelessness during the pandemic further contributed to worsening mental health symptoms with increased feelings of stress, anxiety, and depression for PEH. Behavioral health providers shared that experiencing social isolation worsened mental health conditions and caused some people to return to substance use and fatally overdose. Maintaining open, low-barrier, and equitable access to MOUD during emergencies and ensuring people have regular access to mental health and SUD services during and post-COVID-19 pandemic are necessary. It will continue to be important to assess the impact on substance use even after the COVID-19 pandemic ends. Moving forward, we need to work to maintain as many of the benefits of transitioning services to a virtual environment (e.g., greater reach for therapeutic options and an increased availability of MOUD) as possible while addressing social isolation, and the consequences that come with it.

Our findings also highlight the importance of social connection for individuals in treatment for SUDs. According to a study by Petterson et al. (2020) the United States may experience a projected 75,000 “deaths of despair” due to the isolation and stress caused by the COVID-19 pandemic and warn that if the United States does not begin to implement solutions to the nation’s isolation, pain, and suffering due to COVID-19, then there will be a surge in preventable deaths due to drugs, alcohol, and suicide [20]. This is an opportunity to strategically and universally address the syndemic of homelessness, substance use disorder, and mental health conditions that has combated the United States for years and have increased during the COVID-19 pandemic, while also creating a public health approach to improving mental and emotional well-being. Additionally, as mentioned above, during the COVID-19 pandemic progress was made to secure housing placements for PEH to allow for social distancing. Future work needs to ensure that a sense of community can be maintained even when individuals are transitioned into individual housing opportunities.

The primary limitation of this study was that all interviews were conducted with behavioral health service providers and not directly with clients. Providers have different backgrounds, positions, and caseloads, which may have potentially impacted their perspectives and may not have captured the full breadth of their clients’ experiences during the pandemic. However, providers working with populations disproportionately affected by the pandemic not only have insight into experiences reported by their individual clients, but also offer a broad lens to the issues experienced by the range of clients they serve. Obtaining firsthand information regarding the lived experience of clients is an important next step to complement the data presented here. Additionally, although our sample included providers serving in various behavioral health roles, there was variance in how COVID-19 was being handled across agencies and within regions, impacted by funding and specific policies. Therefore, the findings presented here might not be representative of all experiences across the United States, particularly in rural communities. However, the strengths of this study outweigh these limitations because to our knowledge, it is the first national study of its kind. The findings capture the realities of being a behavioral health provider among disproportionately affected populations amidst the COVID-19 pandemic. This study’s findings provide a basis of what is needed to mitigate the consequences of COVID-19 among these populations.

Additionally, there are methodological limitations of this study. The study team did not calculate or assess quantitative measurement of inter-coder agreement, which could limit the reliability of our results. However, because there were so few coders using a pre-established set of codes who met frequently, it is likely that the exploration of providers’ perceptions and experiences are still descriptive of their true experiences. These themes were also not assessed in light of existing theories, which could impact their transferability to other studies. Despite these limitations, this descriptive, exploratory study offers critical insight to the experiences and perspectives of a critical group of the healthcare and public health workforce.

## 6. Conclusions

Our findings provide additional evidence that the unintended consequences of COVID-19 mitigation strategies, namely social isolation, experienced during the pandemic has been detrimental to mental health and substance use outcomes, especially for PEH. Accessible supportive services for mental health and SUD are critical during emergencies. Furthermore, similar to tracking COVID-19 infections, mental health and substance use disorders need to be measured and disaggregated among demographic and regional categories during emergencies to ensure adequate support and inform interventions.

Mitigating the long-term effects of the pandemic on disproportionately affected populations, particularly people who are experiencing homelessness, substance use, and serious mental illness, requires a strategic, multi-faceted approach via policy, collaborative cross-disciplinary health professionals, and the public. Even after the threat of acquiring COVID-19 subsides, the impact of social isolation on mental health and substance use will continue to be a pervasive issue requiring tailored strategies for PEH and those with SMI or SUD.

## Figures and Tables

**Table 1:** Behavioral Health Service Provider Participant Demographics and Clinic Characteristics, 20 August–14 October 2020.

Attribute	All Participants (*n* = 50)
	*n (%)*
**Gender**	
Female	42 (84%)
Male	8 (16%)
**Age**	
18–34 years	18 (36%)
35–45 years	16 (32%)
46–59 years	11 (22%)
60+ years	5 (10%)
**Race and ethnicity** ^a^	
White, not Hispanic or Latino	31 (62%)
Black or African American, not Hispanic or Latino	10 (20%)
Hispanic or Latino, White race	6 (12%)
American Indian or Alaska Native, not Hispanic or Latino	4 (8%)
Hispanic or Latino, other race	1 (2%)
Other race, not Hispanic or Latino	1 (2%)
Missing	1 (2%)
**Roles**	
Case Manager	10 (20%)
Social Worker	8 (16%)
Therapist or Counselor	7 (14%)
Nurse or Nurse Practitioner	6 (12%)
Outreach Staff	5 (10%)
General/Unspecified Behavioral Health Provider	5 (10%)
Director, Associate Director, or CEO	5 (10%)
Peer Specialist	3 (6%)
Psychiatrist or Psychologist	1 (2%)
**Type of facility or organization** ^b^	
Community health center	32 (64%)
Street team	17 (34%)
Out-patient psychiatric service provider	11 (22%)
Emergency care provider	7 (14%)
Homeless shelter	7 (14%)
Intensive outpatient program	4 (8%)
Other ^c^	4 (8%)
In-patient psychiatric facility	2 (4%)
**Types of services provided** ^b^	
Case management/social service care and referrals	46 (92%)
Outreach and education	43 (86%)
Mental health counseling	40 (80%)
Substance use treatment services	38 (76%)
Primary care	37 (74%)
Evaluations and care planning	34 (68%)
Pharmacotherapies/medication renewal	34 (68%)
Medication for opioid use disorder (e.g., methadone, buprenorphine, vivitrol)	33 (66%)
Rehabilitation or support services (e.g., recovery support groups, AA, NA)	16 (32%)
Other ^d^	11 (22%)
**Clients served** ^e^	
People with a behavioral health related diagnosis	50 (100%)
People experiencing homelessness	49 (98%)
People who use drugs	49 (98%)
People who have experienced or are currently experiencing trauma or violence	48 (96%)
People with serious mental illness that interferes with their ability to perform basic activities of daily living without medication or additional support	44 (88%)

^a^ Participants could select multiple races, so *n* may not add up to 50. However, the denominator used is 50 because that is the total number of people who answered this question. ^b^ Some providers and programs have multiple types of facilities that provide multiple different services as part of larger care networks, so participants could select more than one type of facility and service provided. ^c^ Other types of facilities included: Facilities that specifically provide intensive opioid and other substance use treatment services, and harm reduction facilities. ^d^ Other types of services provided included: Dental, optometry, and podiatry services, specialized care for people living with HIV or AIDS, recreational therapy, and youth-focused services needed for individuals under age 18 years (pediatrics). ^e^ Participants were asked, “What percent of your clients are experiencing homelessness?” “What percent of your clients use drugs?”, etc. Participants that listed a percentage greater than 0 were counted as serving that clientele.

**Table 2:** Themes and Quotes from Behavioral Health Service Provider Interviews on Impact of Social Isolation during the COVID-19 Pandemic on Mental Health, Substance Use, and Homelessness, USA, 20 August–14 October 2020.

Theme or Sub-Theme	Brief Description of Theme	Reflective Quote(s) from Behavioral Health Service Providers
Social Isolation	This cross-cutting theme describes the overall impact of social isolation on client and provider well-being, as well as how social connection is important for health	“[Social isolation is] the worst thing for anybody with mental health or substance abuse”
		“The opposite of addiction is connection.”
		“That’s probably the most prominent, the isolation. Some people would rather risk exposure than be isolated. That’s been pretty common across the board about most of the people I’ve talked to. One of the biggest issues they’re experiencing is the isolation, lack of community, and connection.”
		“For the clients who have been able to get access to isolation hotel rooms, that poses a different set of stressors to access community support and stay engaged. Mental health or substance use conditions deteriorate without having access to either group support or one-on-one like health. We find social support beneficial in general. The social isolation has been a very big stress for clients.”
Return to Substance Use and Increased Substance Use	Providers described the role and influence of social isolation on re-initiating or increasing substance use. This theme captures providers’ perspectives on how COVID-19 and social isolation changed substance use patterns.	“They don’t have that interaction that they had before. Before we had COVID-19, we had medication assisted treatment, as well as groups, and more access physically to their counselor. So that one-two combination, it’s proven successful in helping people in their recovery. They don’t have that now.”
		“So we noticed that some of the clients are relapsing, and they’ll say “I’m having a little anxiety, I’m not able to come into the clinic as much as I want to, because of quarantine and shelter in place. I don’t have a support group to talk to. I don’t have minutes on my phone anymore. I’m getting anxious. I’m getting nervous, the thoughts are coming back” and then you start seeing the [return to substance use] signs. And it all boils down to, you know, they say “I’m lonely.”“
		“I think that relapse is a big thing. You spend 10 years of doing a specific drug, or way of life or living or several drugs, and then trying to get off and then based off of the world shifting in such, what I call madness, it kind of gives you a reason to relapse and go backwards. So that’s been a big issue with relapsing. It’s an up and down and up and down thing, stronger than it already has been because our clients are facing homelessness, addiction, and mental health. So relapse is a big thing, and out here on the streets, drugs are, clearly they’re here. But I feel like a wave of more [drugs] have come in, so it’s been kind of chaotic”
		“I think that [social isolation] increases stress tremendously, and people seek ways to manage stress. And that can involve increased substance use, increased risk behaviors or increase possibility of self-harm. It’s when people don’t feel like there’s choices, it’s harder to find ways to move forward.”
		“People are tending to use in a little bit more risky way than they had been”
Changes in Overdose Risk and Rates	Providers described the increased risk for overdose as a result of social isolation. Recommendations to socially isolate or distance from others contradicted recommended strategies for overdose prevention, leaving many clients unsure what to do. They also described changes in overdose patterns.	“Being alone, more people have overdosed. The messaging about need[ing] to isolate didn’t do a good job of taking the risks involved into account.”
		“[Isolation from others] is the exact opposite of what we need for an opioid epidemic.”
		“I’ve had many more clients die. No one has died of coronavirus, but from overdose, especially because people are alone a lot more.”
		“I think people are using more substances. I think they’re using alone. We have an increase of I think 50% more deaths in the homeless population due to overdosing. So there’s been some things that have been available to them that they never had before, and I think they’re also suffering in ways that they weren’t before.”
		“I think probably the biggest thing is that being alone, more people have overdosed. There’s been a fair number of overdoses at the hotel rooms, like fatal overdoses. Probably non-fatal overdoses as well. But I know less about those. And I think people were probably not adequately prepared for that risk. I think that the messaging about ‘you all need to isolate’ didn’t do a good job of taking the risks involved into account.”
		“So people reported a lot of feelings of abandonment and we did have two people pass away from overdose.”
		“We’ve had a significant increase in our overdose deaths and I do think that it is a result of a change in drug supply. There’s not as many dealers around because people are isolating and quarantining.”
Access to Medications for Opioid Use Disorder	Providers described beneficial policy changes to facilitate better access to opioid use disorder treatment.	“I think it’s the way we would provide medically assisted treatment in a shared medical visit setting. So, it would be in a group setting and we would kind of do a group accountability with a multi-disciplinary team. We’re not doing that anymore, so a lot of our patients are relapsing because they don’t have the social support component of the group modality. So that’s been really difficult for our patients. Although it’s increase[d] accessibility to Buprenorphine, it’s decreased the support services around it, so a lot more patients are relapsing.”
		“For people experiencing substance use disorders, the changes in substance use services have been very dramatic. In the clinic that I work at, we’ve been able to provide people with 30 days worth of Suboxone scripts which, I think, has been beneficial. But that also comes with a cost of not having people have the same access to groups where they’re able to engage with other people working on their recovery and to have the social supports that are really crucial in being able to address addiction.”
		“I’ve talked about isolation being one of the biggest [challenges], but I imagine for the clients that I’ve spoken with, getting access to medication is also a concern. Often the insurance companies don’t want to provide for more than a 30-day scrip, and the pharmacy that we’ve worked with in the past had real challenges in being able to help clients get 90-day supplies with the insurance approval. So, that’s something that I know has been a concern.”
Exacerbation of Mental Illness	Providers described how being socially isolated contributed to increased symptoms of mental health illnesses.	“If you have a serious mental illness, [isolation] seems to magnify whatever illness that person struggles with—depression, anxiety, psychosis—isolation is really detrimental.”
		“For a good chunk of the people that I work with, who struggle with depression or anxiety, they still struggle with being alone or isolating themselves for that chunk of time. Because it makes their symptoms worse. It can make their depression worse, that can make their anxiety symptoms worse, which obviously is just super uncomfortable, and who would want to make themselves feel worse when they’re already struggling? I would say that’s probably the biggest thing.”
		“A lot of people with mental illness have a routine or have a place in the community where they belong. Maybe it’s a coffee shop or they go into the library and use the computer or a community center. All those things are shut down. Talking to my patients, there’s a lot of feeling lost, that they don’t belong anywhere, they’re not welcome anywhere. Those routines were kind of gone and I think a lot of people’s mental health suffered. A lot of people are reporting increased depression and anxiety, not sure where to go during the day.”
		“There’s a lot of skepticism, paranoia, [and] delusions. And so, there’s a lot of delusions all the time about being watched, or being targeted, or like the government is watching, or they’re listening, kind of things like that. And so, something like this really, really kicked that into high gear for a lot of clients.”
		“There has been such a feeling of fear and apprehension and mistrust that we’ve had to overcome to really let people know that we’re just here to try to take care of them. When the pandemic first—I’m thinking back to April and May [2020], we had the National Guard here with us at our emergency shelter and the hotel. And they were very helpful. But there was a great deal of fear seeing these soldiers at our sites. And I had people who were homeless tell me that they thought that we, meaning the service providers, were going to lock them all up in a concentration camp.”
Navigating Homelessness while Experiencing Mental Illness or Substance Use Disorder	This theme captures providers’ descriptions of how responses to homelessness during COVID-19 helped some receive housing, and how being housed contributed to further social isolation. People had to make complex decisions about housing situation and mental well-being.	“Folks’ anxiety has definitely increased. I think there’s been a significant amount of people whose depressive symptoms have also increased, given the fact that they have been locked into a certain place, and really don’t have any good options or capacity to get out. If they do, they’re oftentimes not able to return to that situation, or return to that shelter, which gives them an even tougher predicament. If they do leave, they are out there in the world, most likely unsheltered, and having significant safety concerns with that, and not having the capacity or access to food, to water, to the things that the shelter might be -- that the shelters are offering.”
		“Some shelters started requiring that people got a COVID test or they couldn’t come back to the shelter. And that was really stressful for some because they didn’t know where to get the test. And they were so stressed out because they had nowhere to go. We had a guy crying in our lobby. He said I’m too old for this. I can’t stay on the street. I’m too old. And none of the shelters will take me back because I haven’t had the COVID test. And he was just really, really depressed and distraught.”
		“Housed clients are doing a better job of social distancing, but then experiencing higher levels of isolation, boredom, and depression”
		“Because it’s different. Even if the sheltered life is not the best life, you have distractions, you have other people around that are friends you have social things that you see, you have interactions with people. And when you’re alone, you’re just kind of alone with your thoughts and to think about things, and reflecting on your life and look around and say like, “I have this thing I always wanted, but I just feel so alone because I’ve lived in this environment where there was always some kind of stimulation going on, and now I don’t have anything. What do I do with that?””
		“A small percent [of street homeless] were able to get in some temporary housing that would not have happened without COVID”

## Data Availability

The data presented in this study are available on request from the corresponding author. The data are not publicly available due to privacy reasons.

## Supplementary Materials

The following supporting information can be downloaded at: https://www.mdpi.com/article/10.3390/ijerph191912120/s1, File S1. Impact of Social Isolation During the COVID-19 Pandemic on Mental Health, Substance Use, and Homelessness: Interview Guide.
