# Supplementary material for: Impact of Social Isolation during the COVID-19 Pandemic on Mental Health, Substance Use, and Homelessness: Qualitative Interviews with Behavioral Health Providers
Source: Int J Environ Res Public Health. 2022 Sep 25;19(19):12120. doi: 10.3390/ijerph191912120 (PMC9566547; doi:10.3390/ijerph191912120)
Supplement: Supplementary file 1 [file ijerph-19-12120-s001.zip › ijerph-1882009-supplementary.pdf]

<u>Impact of Social Isolation During the COVID-19 Pandemic on Mental Health, Substance Use, and Homelessness: Interview Guide</u>

Provider location (City, State):
Date:
Interviewer Name:
Notetaker Name:
Participant ID:

(Instructions to notetaker: Participant ID: notate as interviewer's initials_MMDD_TTTT (ex. VF_0823_1830)). The notetaker will manage the recording and will start recording at the end of the verbal consent (notated below). Please refer to the notetaker document for further guidance. Interviewer please make sure you have the participant ID available before the interview.)

Interviewer: Good morning/afternoon. I am [NAME] and I am joined by [NAME] who will be taking notes during our discussion. This is part of a voluntary project being conducted by the Centers for Disease Control and Prevention in collaboration with National Healthcare for the Homeless. Thank you for taking the time to speak with us today about the impacts of behavioral health issues on COVID-19 risks and disruptions in behavioral health services caused by COVID-19 among people experiencing homelessness.

I will now go over a consent form with you prior to starting the interview.

These interviews are being conducted to inform new or best practices for the provision of behavioral health services for people experiencing homelessness.

We would like to hear your opinions about the topics we discuss. There are no right or wrong answers to any of the questions we ask today. You are the expert on your experience, and your thoughts and opinions are greatly valued and appreciated. We encourage you to speak openly about your opinions and experiences. Anything that you share will be kept private, and your responses will be grouped with answers from other participants. No identifiable information will be known after responses are grouped.

With your permission, I would like to audio record this conversation to ensure that the notes we take are accurate and comprehensive. The recording will be stored securely until it is transcribed, after which it will be destroyed.

All information collected from these interviews will be housed on a secure drive on the CDC network only accessible to project members and will be shared only in aggregate form. We will not include names in any reports. The interview should take approximately 60-90 minutes. If you would like to skip a particular question or would like to stop the interview at any time, please let me know.

As a show of gratitude for your time and input, we will be providing you with a gift card in the amount of $100.

Do you have any questions before we begin? *Notetaker documents any questions.*

We will start the recording now.

Notetaker: Start the recording.

***Interviewer: State Interviewer name, date, time, and Participant ID*.**

To have it on record we would like to start by asking:

Do you agree to participate in this interview?

○ Yes

○ No

Do we have your permission to record this interview?

○ Yes

○ No

Thank you!

<u>If participant filled out the REDCap (Research Electronic Data Capture) survey:</u>

<u>Thank you for filling out the REDCap survey prior to the interview. We can proceed to the qualitative portion of the interview.</u>

Warm-up: I will briefly go over the structure of the interview.

The qualitative interview consists of questions concerning behavioral health issues and services provided by your facility/organization in relation to COVID-19. There are three parts that cover: 1) the impact of behavioral health issues on COVID-19 prevention recommendations; (2) changes to behavioral health services provided by your organization as a result of COVID-19; and (3) potential challenges and unexpected benefits that your organization or clients have faced since the start of the pandemic.

<u>Pre-Discussion Questionnaire (Facility, Staff, & Client Characteristics).</u>

Skip if person has filled this section out prior to the start of the interview on RedCAP.

Only read if REDCap not completed: Prior to the qualitative portion, we will go over a questionnaire consisting of 14 questions that include individual demographics, facility, staff and client characteristics. We have provided you with the quantitative questionnaire prior to the interview. Please refer to this now if possible, to facilitate answering the questions. We will read out the responses if you are unable to refer to the questions.

***Interviewer: Read the questions and go over the options one at a time.**

Notetaker: To be entered here first for ALL participants (highlight the responses in yellow). The notetaker will enter into REDCap following the interview if not already completed by the provider

1) Sex/Gender: How to you identify?
○ Male
○ Female
○ Transgender or Gender-nonconforming
○ Decline

2) What is your Age?

- ○ 18-34
- ○ 35-45
- ○ 46-59
- ○ 60+
- ○ Decline

3) What is your Race (check all that apply)?

- ☐ American Indian or Alaska Native
- ☐ Asian
- ☐ Black or African American
- ☐ Native Hawaiian or Other Pacific Islander
- ☐ White
- ☐ Other
- ☐ Decline

4) What is your Ethnic affiliation?

- ☐ Hispanic
- ☐ Non-Hispanic
- ☐ Decline

5) What is your job title? ____

6) How long have you been in this role at your current organization? ________ (use 0.1 to 0.11 to notate 1 to 11 months (if for example 5 years and 11 months, put 5.11 or if 10 months, put 0.10))

7) What is the type of facility or organization where you work (check all that apply)?

- ☐ In-patient psychiatric facility
- ☐ Intensive outpatient program
- ☐ Out-patient psychiatric service provider
- ☐ Emergency care provider
- ☐ Street team
- ☐ Homeless shelter
- ☐ Community health center
  - If yes, Health Care for the Homeless? ○Yes ○ No
- ☐ Other, specify: ____________________________

8) What type(s) of services does your facility or organization provide? Check all that apply.

- ☐ Primary care
- ☐ Mental health counseling
- ☐ Substance use treatment services
- ☐ Outreach and education
- ☐ Evaluations and care planning
- ☐ Case management/social service care and referrals
- ☐ Pharmacotherapies/medication renewal
- ☐ Medication for opioid use disorder (e.g., methadone, buprenorphine, Vivitrol)

☐ Rehabilitation or support services (e.g., recovery support groups, AA, NA)
☐ Other, specify: ______________________________

9) Does your organization provide services at a single site or multiple sites?
○ Single site
○ Multiple sites

10) What types of staff work at your facility or organization? Check all that apply.
☐ Physicians
Psychiatrist ○ Yes ○ No
☐ Physicians' assistants
☐ Nurse practitioners
☐ Nurses
☐ Substance use counselors
☐ Psychologists
☐ Case managers
☐ Social workers/therapists
☐ Community health workers
☐ Peer counselors
☐ Recovery coaches
☐ Other, specify: ______________________________

11) What types of clients do you serve? Check all that apply.
☐ People experiencing homelessness
If yes: about what % of clients served have experienced or are currently experiencing homelessness? ____________________
☐ People who use drugs (e.g., marijuana [legal or illicit], cocaine, heroin or other opioids [including misuse of prescription opioids], methamphetamine, etc.)
If yes: about what % of clients use drugs? ____________________
If yes: about what % have a formal substance use disorder diagnosis? _______________
☐ People with a behavioral health related diagnosis
If yes: about what % of clients have a behavioral health related diagnosis? ____________
☐ People with serious mental illness that interferes with their ability to perform basic activities of daily living without medication or additional support
If yes: about what % of clients have a serious mental illness? ____________
☐ People who have experienced or are currently experiencing trauma or violence
If yes: about what % of clients experienced or are currently experiencing trauma or violence? ____________

12) What are the most commonly accessed or utilized services by your organization's clients experiencing homelessness?
____
____

13) What would you say are the 2-3 most common behavioral health conditions among clients experiencing homelessness at your facility or organization?

____

____

14) What are the most common drugs used among your clients experiencing homelessness?

____

____

<u>**Qualitative Discussion Guide**</u>

Throughout the interview, we are going to ask questions relating to three specific populations-persons experiencing homelessness, persons with substance use disorders and persons with mental illness. For each group, think of any differences that you think should be highlighted.

Part 1 – Impact of Behavioral Health Issues on COVID-19 Prevention Recommendations

**Pivot statement –** Now, I would like to talk about the impact that behavioral health issues among people experiencing homelessness may have on their adherence to COVID-19 prevention recommendations. We are going to ask you a series of questions related to social distancing, masks, proper hand hygiene, and isolation and quarantine recommendations. For these questions, you can discuss what is occurring in your facility as well as the community.<br>Some of the follow up questions may sound repetitive, but that is because we are trying to get information for all 3 groups if possible. i.e. persons experiencing homelessness, persons with substance abuse disorder, and persons with severe mental illness.

Interviewer note: It is not necessary to ask all probes if a particular probe is not indicated or already has been covered. It is also not necessary to ask all questions if an answer has been provided by the interviewee under a different question. The order of questions can be adjusted if this helps with interview flow. Can add additional probes to ask about behavioral (or structural barriers) if the interviewee is only focusing on one.

1) What have you observed or have your clients shared regarding any behavioral barriers to adhering to social distancing recommendations?
    - Probe: What adherence issues have been unique to people experiencing homelessness, if any?
    - Probe: From your observations, does the experience of homelessness itself create additional adherence challenges, separate from behavioral health issues?
    - Probe: What adherence issues have been unique to clients receiving treatment for substance use disorders, if any?
    - Probe: What adherence issues have been unique to clients with serious mental illness, if any?
    - Probe: How do you think these adherence challenges could be mitigated?

2) What have you observed or have your clients shared regarding any behavioral barriers to wearing a mask (for example, cloth face covering)?
   - Probe: What adherence issues have been unique to people experiencing homelessness, if any?
   - Probe: From your observations, does the experience of homelessness itself create additional adherence challenges, separate from behavioral health issues?
   - Probe: What adherence issues have been unique to clients receiving treatment for substance use disorders, if any?
   - Probe: What adherence issues have been unique to clients with serious mental illness, if any?
   - Probe: How do you think these adherence challenges could be mitigated?

3) What have you observed or have your clients shared regarding any behavioral barriers to adhering to practicing proper hand hygiene?
   - Probe: What adherence issues have been unique to people experiencing homelessness, if any?
   - Probe: From your observations, does the experience of homelessness itself create additional adherence challenges, separate from behavioral health issues?
   - Probe: What adherence issues have been unique to clients receiving treatment for substance use disorders, if any?
   - Probe: What adherence issues have been unique to clients with serious mental illness, if any?
   - Probe: How do you think these adherence challenges could be mitigated?

4) What have you observed or have your clients shared regarding any behavioral barriers to adhering to isolation and quarantine recommendations?
   - Probe: What adherence issues have been unique to people experiencing homelessness, if any?
   - Probe: From your observations, does the experience of homelessness itself create additional adherence challenges, separate from behavioral health issues?
   - Probe: What adherence issues have been unique to clients receiving treatment for substance use disorders, if any?
   - Probe: What adherence issues have been unique to clients with serious mental illness, if any?
   - Probe: How do you think these adherence challenges could be mitigated?

Part 2 – Changes to Behavioral Health Services Provided

**Pivot statement –** Now, I would like to talk about the changes to behavioral health services provided by your organization or at your facility as a result of COVID-19.

5) In what ways have you made changes to your behavioral health services since the start of the COVID-19 pandemic?

- Probe: What are the reasons for changes to your services (e.g., offices closed, need to limit client contact, lack of PPE)?
- Probe: How do the current behavioral health services compare to the services provided before the pandemic?
- Probe: How and where are the current behavioral health services being provided? How has the delivery method changed (e.g., in-person, telemedicine)?
- Probe: How have behavioral health services changed for clients experiencing homelessness?
- Probe: How have behavioral health services changed for clients with serious mental illness?
- Probe: Has your facility or organization increased the number of take-home doses, changed procedures, or otherwise lowered barriers to access medications such as methadone or buprenorphine as a result of the pandemic?

6) In what ways has client engagement or interaction with behavioral health services at your organization or facility changed since the start of the COVID-19 pandemic?
    - Probe: What are the most commonly accessed or utilized behavioral health services now compared to before the pandemic among clients experiencing homelessness?
    - Probe: How have client-specific changes, such as relocation of clients experiencing homelessness to non-congregate sites, affected their engagement with your behavioral health services?
    - Probe: How has client engagement and distribution of medications changed among clients receiving treatment for substance use disorder?
    - Probe: How has engagement changed among clients with serious mental illness?

Part 3 – Challenges and Unexpected Benefits

**Pivot statement –** For this next part of the interview, I would like to talk more about the challenges and unexpected benefits that **you or your facility** may have faced since the start of the COVID-19 pandemic. **I will ask you about issues your clients have faced later.**

7) What, if any, barriers or challenges have you or your facility faced with continuing to provide behavioral health services to your clients since the beginning of the COVID-19 pandemic?
    - Probe: Provide an example of a challenge you have faced since the start of the pandemic.
    - Probe: Have staff identified knowledge gaps or need for additional training during the pandemic?

8) Have there been any changes that led to unexpected benefits or helped facilitate care at your organization or facility? For example, has a certain change created the opportunity to provide services to more clients?
    - Probe: Provide an example of a new approach, an opportunity, or positive experience you or other staff have faced.
    - Probe: What tools or resources have your clinicians accessed to begin providing new substance abuse treatment to clients during the pandemic? For example, applying for a DATA 2000 waiver to start buprenorphine treatment, or providing take-home medications for opioid use disorder?

9) How have local, state, or federal policies or actions taken in response to COVID-19 contributed (if at all) to challenges or benefits you have faced or are facing in your work?
    - Probe: Some policies or actions to consider may include: stay at home orders, reopening plans/limitations, school or childcare closures, availability of PPE, requests for motel vouchers, standing up or maintaining isolation/quarantine facilities, or others.
    - Probe: How have changes in infection prevention and control influenced or impacted the challenges or benefits you have faced in your work?

Note to interviewer: **For Q10, if the interviewee has covered telemedicine extensively in other questions, you can ask** "Since you already mentioned telemedicine previously, is there anything else you would like to add in terms of challenges or benefits of telemedicine?" **or if already covered exhaustively, you can skip this question.**

10) If you switched from in-person services to telemedicine (either via video or audio-only), have you encountered any challenges or benefits with this switch?
    Only ask if they did not switch: If you did not switch services from in-person to telemedicine, have you encountered any challenges or benefits continuing in-person services?

    If yes (to either), what are the specific challenges or benefits you have encountered?
    - Probe: How has telehealth changed your clinical approach, if at all?
    - Probe: What has been the impact on clients experiencing homelessness?
    - Probe: What has the impact been on clients receiving treatment for substance use disorders?
    - Probe: What has been the impact on clients with serious mental illness?

**Pivot statement –** Now that you've told me about you and your facility, I want you to think about challenges and unexpected benefits that **your clients** may have faced since the start of the COVID-19 pandemic.

11) What challenges, if any, do you think your clients have faced in accessing behavioral health services since the start of the COVID-19 pandemic? These may include but are not limited to: challenges accessing care or medication, lack of transportation, concerns over going out, changes in drug supply, or additional stressors caused by the pandemic that could lead to exacerbation of psychiatric symptoms, etc.
    - Probe: Provide an example of a challenge a client has faced.
    - Probe: Are any of these challenges unique to clients experiencing homelessness?
    - Probe: Are any of these challenges unique to clients receiving treatment for substance use disorders?
    - Probe: Are any of these challenges unique to clients with serious mental illness?
    - Probe: How do you think these challenges could be mitigated on a programmatic or policy level?

12) Have any changes during COVID-19 led to unexpected benefits or helped facilitate care for your clients?
    - Probe: For example, has a certain change in services created the opportunity for clients to access medication or treatment more easily, or resulted in more resilience among clients?
    - Probe: Have clients experiencing homelessness described any changes that have been beneficial?
    - Probe: Have clients who use drugs described any changes that have been beneficial?
    - Probe: Have clients with serious mental illness described any changes that have been beneficial?

Pivot statement – We're almost to the end of the interview now; I just have a few more questions about your perspective.

13) Have you seen more of your clients become homeless during the COVID-19 pandemic?
    - Probe: If yes, what do you think are the reasons for this?

14) As a result of the COVID-19 pandemic, have you seen clients who are experiencing homelessness face any other particular struggles that have impacted their behavioral health that we have not discussed?

15) As a result of the COVID-19 pandemic, have you seen any clients experiencing homelessness have new opportunities for shelter or housing, such as hotel placements or expedited permanent housing placements?
    - Probe: How have these new shelter arrangements affected their behavioral health?
    - Probe: How has your community made new housing opportunities available?

16) We have talked about a lot of different elements related to behavioral health impacts affecting adherence to COVID-19 prevention recommendations, and what challenges and opportunities you and your clients are facing. Is there anything else you would like to share, or that you think is important that I have not asked you about?

That concludes our interview. Thank you again for your time and for sharing your perspective as a behavioral health service provider. Your responses will be analyzed together with the responses of other behavioral health service providers and compiled into a report, which will help inform CDC's guidelines.

Stop Recording

You will be sent an email link for a $100 Amazon gift card within a month from National Health Care for the Homeless Council. We will follow up with you to ensure you received it.

Please do not hesitate to reach out if you have any questions.

Note: Please use the naming convention *interviewer's initials_MMDD_TTTT* (ex. VF_0823_1830).*mp4* when saving and storing your recordings in the must drive. *Note: This is the same naming convention as the participant ID.*
